# Intestinal Barrier Integrity in Heat-Stressed Modern Broilers and Their Ancestor Wild Jungle Fowl

**DOI:** 10.3389/fvets.2020.00249

**Published:** 2020-05-07

**Authors:** Travis W. Tabler, Elizabeth S. Greene, Sara K. Orlowski, Joseph Z. Hiltz, Nicholas B. Anthony, Sami Dridi

**Affiliations:** Department of Poultry Science, University of Arkansas, Fayetteville, AR, United States

**Keywords:** chicken, intestinal integrity, growth rate, tight junction, gap junction

## Abstract

High environmental temperature has strong adverse effects on poultry production, welfare, and sustainability and, thereby, constitutes one of the most challenging stressors. Although colossal information has been published on the effects of heat stress on poultry productivity and gut health, the fundamemntal mechanisms associated with heat stress responses and intestinal barrier function are still not well defined. The aim of the present study was, therefore, to determine the effects of acute (2 h) heat stress on growth performance, gut integrity, and intestinal expression of heat shock and tight junction proteins in slow- (broilers of the 1950's, ACRB), moderate- (broilers of 1990's, 95RAN), rapid-(modern broilers, MRB) growing birds, and their ancestor wild jungle fowl (JF). Heat stress exposure significantly increased the core body temperature of 95RAN and MRB chickens by ~0.5–1°C, but not that of JF and ACRB compared to their counterparts maintained at thermoneutral conditions. Heat stress also depressed feed intake and increased serum fluorescein isothiocyanate-dextran (FITC-D) levels (*P* < 0.05) in modern broilers (95RAN and MRB) but not in JF and ACRB, indicating potential leaky gut syndrome. Molecular analyses showed that heat stress exposure significantly up regulated the duodenal expression of occludin (*OCLN)* and lipocalin (*LCN2)* in ACRB, zonula occludens (*ZO-2)*, villin1 *(VIL1)*, and calprotectin (*CALPR)* in 95 RAN, and only *CALPR* in MRB compared to their TN counterparts. In the jejunum however, heat stress down regulated the expression of PALS1-associated tight junction protein (*PATJ)* in ACRB, 95RAN, and MRB, and that of cadherin1 (*CDH1)* in MRB. In the ileum, heat stress significantly down regulated the expression of *OCLN* in 95 RAN, *ZO-1* in MRB, gap junction protein alpha1 (*GJA1)* in JF, and *VIL1* in ACRB compared to their TN counterparts. In summary, this is the first report, to our knowledge, showing that tight junction protein expression is environmental-, genotype-, and intestinal segment-dependent and identifying molecular signatures, such as *CDH1, CALPR*, and *ZO-1*, potentially involved in leaky gut syndrome-induced by heat stress in MRB.

## Introduction

Poultry genetic selection for high growth rate and enhanced muscle development, driven by economic demand over the past 80 years, has made spectacular progress in term of breast yield, feed efficiency, and reduction of market age (1). Indeed, compared to broiler of the 1950's, a modern broiler of today achieves ~400% increase in body weight arising mainly from breast muscle during 56 day period (2). These progresses make broiler (meat-type) chickens the most efficient and inexpensive food source (3) with a global world's broiler meat production amounted to about 97.8 million metric tons in 2019 (4). Although broiler production supports the livelihoods and food security of billions of people (5), it is facing several significant challenges from a steep projected 73% increase in global demand for animal proteins (meat and eggs) by 2050 which is steered by growing human population (6), and the need to adapt to the threats posed by climate changes particularly global warming (7–9).

Unusual warm season and temperature anomalies have increased markedly in the past decades and are affecting biological systems including livestock animals, insects, and crops (9). Due to their high metabolic activity and lack of sweat glands, modern broilers are particularly sensitive to heat stress. The strong adverse effects of heat stress on growth, feed intake, feed efficiency, meat yield and quality, welfare, and mortality are well documented in broilers (10–20). Emerging evidence indicates that heat stress alters also the gut morphometry (lower crypt depth, mucous area, and villus height) which in turn affect nutrient absorption (21). Recent study has shown that heat stress increases FITC-D levels in chicken serum indicating an increased intestinal permeability and leacky gut syndrome (22). Koch and co-workers have shown that heat stress directly impairs gut integrity and disturbs the immune cell populations in bovine intestine (23). This breakdown of the intestinal barrier has been shown to allow opportuinistic bacteria to enter circulation resulting in infections such necrotic enteritis (24), femoral head necrosis, and bacterial chondronecrosis with osteomyelitis (25). Although tremendous information has been published on the effects of heat stress on productivity in poultry, the fundamemntal mechanisms associated with the effects of heat stress and rapid growth rate on intestinal barrier function are not known. The aim of the present study was, therefore, to determine the effects of acute heat stress on growth performance, blood FITC-D concentrations, and intestinal expression of heat shock and tight junction proteins in broilers of the 1950's (26, 27), 1995 (28), 2015 (29), and their ancestor jungle fowl (29).

## Materials and Methods

### Chicken Populations

The broiler chickens involved in this trial were hatched from eggs collected at the University of Arkansas research farm and consist of four research lines; three of which represent the commercial broiler chicken of the 1950s (Athens Canadian Random Bred, ACRB) which is characterized by a slow-growth (26, 27), 1995 (95RAN) which is a moderate-growing line that has the genetics of 7 male and 6 female commercial broiler lines available in the mid-1990s (28), and 2015 modern random bred (MRB) which is composed of broiler packages offered by three broiler genetics companies and have been blended homogenously after many generations of random mating (29), and the fourth is the South East Asian jungle fowl, the wild-type ancestor (JF) (29). All populations are maintained at the University of Arkansas research farm under close care and supervision. Each generation is randomly mated with the exception of full and half sibling pairings. The study was conducted in accordance with the recommendations in the guide for the care and use of laboratory animals of the National Institutes of Health and the protocols were approved by the University of Arkansas Animal Care and Use Committee under protocols 18083 and 16084.

### Birds and Diets

Day-old broiler chicks from the four chicken populations were hatched at the University of Arkansas, vent-sexed, individually wing-banded with a number and barcode, and housed in environmentally controlled chambers in the Poultry Environmental Research Laboratory at the University of Arkansas. Male chicks were separated by line and placed into twelve environmental chambers with each chamber consisting of two equally sized pens allowing for triplication of a 4 x 2 factorial design. Twenty-five male chicks of the same line were randomly placed in each pen and kept at an approximate density of 0.5 m^2^ per bird in all pens. All birds had *ad libitum* access to feed and fresh water. During the first week, birds were provided with a 23 h light/1 h dark lighting program and subsequently a 20 h light/4 h dark lighting program throughout the remainder period (day 8 to 56) of the trial. Commercially available starter and finisher diets, formulated to meet or exceed NRC recommendations (30), were fed from 0 to 28 days and from 29 to 56 days, respectively. Rearing temperature gradually decreased from 32°C on days 1 to 3, to 31°C on days 4 to 6, 29°C on days 7 to 10, 27°C on days 11 to 14, and 24°C for day 15 through day 28; on the morning of day 29 the birds were subjected to one of two environmental conditions: thermoneutral (TN, 24°C) or acute (2 h) heat stress (HS, 36°C).

### Samples Collection

Feed (FI), body weight (BW), core body temperature, pen temperature and relative humidity were recorded. Birds used for biological sampling were equipped with Thermochron temperature sensors (iButton, DS1922L, Maxim, CA), which recorded core body temperature. Blood samples were collected for blood parameters, and small intestine segments (duodenum, jejunum, and ileum) were harvested for molecular and biochemical analyses as described below. The tissues were snap frozen in liquid nitrogen and stored at −80°C until analysis.

### Determination of Serum Fluorescein isothiocyanate-dextran (FITC-D) Levels

Serum FITC-D concentrations were determined as previously described (22). Briefly, 1 h before blood collection, 6 birds from each group were orally gavaged with FITC-D (8.32 mg/kg, MW 3-5 kDa, Sigma-Aldrich, St. Louis, MO). Blood was collected l h post gavage and fluorescence was measured at an excitation wavelength of 428 nm and emission wavelength of 528 nm using the Synergy HT multi-mode micro plate reader (BioTek,Winooski, VT).

### RNA Isolation, Reverse Transcription, and Quantitative Real-Time PCR

One μg of total RNA was extracted from the sampled tissues using Trizol reagent (ThermoFisher Scientific, Rockford, IL) in accordance with the manufacturer's recommendations. RNA concentration was determined by Take 3 micro volume plate and the Synergy HT multi-mode microplate reader (BioTek, Winooski, VT). RNA quality and integrity were assessed using the ratio of absorbance (260/280) and 1% agarose gel electrophoresis. RNAs were then treated with DNAse, and reverse transcribed via qScript cDNA SuperMix (Quanta Biosciences, Gaithersburg, MD). The cDNA was then amplified by real-time quantitative PCR (Applied Biosystems 7500 Real-Time PCR system) with Power SYBR green Master Mix (ThermoFisher Scientific, Rockford, IL) in a total volume of 20 μL per reaction. Oligonucleotide primers specific for chicken inflammation-, gap junction-, and tight junction-related genes were used (Table 1). Primers for heat shock proteins (HSP60/70/90) were previously described (13, 31). The qPCR cycling conditions were 50°C for 2 min, 95°C for 10 min followed by 40 cycles of a two-step amplification program (95°C for 15 s and 58°C for 1 min). At the end of the amplification, melting curve analysis was applied using the dissociation protocol from the Sequence Detection system to exclude contamination with unspecific PCR products. The PCR products were also confirmed by agarose gel and showed only one specific band of the predicted size. For negative controls, no RT products were used as templates in the qPCR and verified by the absence of gel-detected bands. Relative expressions of target genes were determined by the 2^−Δ*ΔCt*^ method (32) and JF at TN conditions was used as calibrator.

**Table 1:** Oligonucleotide real-time qPCR primers.

**Gene**	**Accession number^a^**	**Primer sequence (5' → 3')**	**Orientation**	**Product size (bp)**
OCLN	NM_205128	CGCAGATGTCCAGCGGTTA	Forward	59
		GTAGGCCTGGCTGCACATG	Reverse	
ZO-1	XM_015278975	GGGAACAACACACGGTGACTCT	Forward	80
		AGGATTATCCCTTCCTCCAGATATTG	Reverse	
ZO-2	NM_204918	GCAATTGTATCAGTGGGCACAA	Forward	69
		CTTAAAACCAGCTTCACGCAACT	Reverse	
ZO-3	XM_015299757	CAAAGCAAGCCGGACATTTAC	Forward	63
		GTCAAAATGCGTCCGGATGTA	Reverse	
JAMA	EF102433	TCACCTCGGAGACAAAGGAAGT	Forward	60
		ACGCAGAGCACGGGATGT	Reverse	
GJA1	NM_204586	TGGCAGCACCATCTCCAA	Forward	56
		GGTGCTCATCGGCGAAGT	Reverse	
PATJ	XM_015291147	GGATCCAGCAACGTGTCCTATT	Forward	114
		GCATCCAGTGGAGTGTCTTTCC	Reverse	
CDH1	AF098472	GGGAGCGCGTTGCCTACTA	Forward	57
		GAGGGCTGCCCAGATCTGA	Reverse	
Cx45	M35044	TCCACCTTCGTTGGCAAAA	Forward	58
		TCAGAACGATCCGAAAGACGAT	Reverse	
VIL1	NM_205442	TGCCGGTGCCCACTAAAA	Forward	63
		TCGACAGCAGCACGTAGCA	Reverse	
LCN2	AY082334	TGCAGCTTGCAGGGAGATG	Forward	69
		GCTTCTTGTCCTTGAACCAGTTG	Reverse	
CALPR	XM_424012	GCTGGAGAAAGCCATTGATGTC	Forward	61
		CCCCTCCCGTCTCGAGTAC	Reverse	
18S	AF173612	TCCCCTCCCGTTACTTGGAT	Forward	60
		GCGCTCGTCGGCATGTA	Reverse	

a *Accession number refer to Genbank (NCBI)*.

*CALPR, calprotectin; CDH1, cadherin 1; Cx45, connexin 45; GJA1, gap junction protein alpha 1; JAMA, junctional adhesion molecule A; LCN2, lipocalin 2; OCLN, occludin; PATJ, PALS1-associated tight junction protein; VIL1, villin 1; ZO-1/2/3, zonula occludens*.

### Statistical Analysis

Data were analyzed by two-way ANOVA, with chicken population (JF, ACRB, 95RAN, MRB) and environmental condition (TN, HS) as the main effects. Core body temperature data were analyzed using two-way repeated-measures ANOVA with time as the repeated measure and treatment (TN vs. HS) as factors. If ANOVA revealed significant effects, the means were compared by Tukey's multiple range test using the Graph Pad Prism version 6.00 for Windows (Graph Pad Software, La Jolla California, USA). The experimental model was as follows: yijk = μ + αi + βj + (αβ)ij + εijk, where μ is the general mean, αj is the environmental effect (E), βj is the effect of chicken population (genotype, G), (αβ)ij is the ExG interaction effect, and εijk is the experimental error. Differences were considered significant at *P* < 0.05. Data are expressed as the mean ± SEM.

## Results

### Effects of Heat Stress on Growth Performances and Core Body Temperature

**Figure 1:**
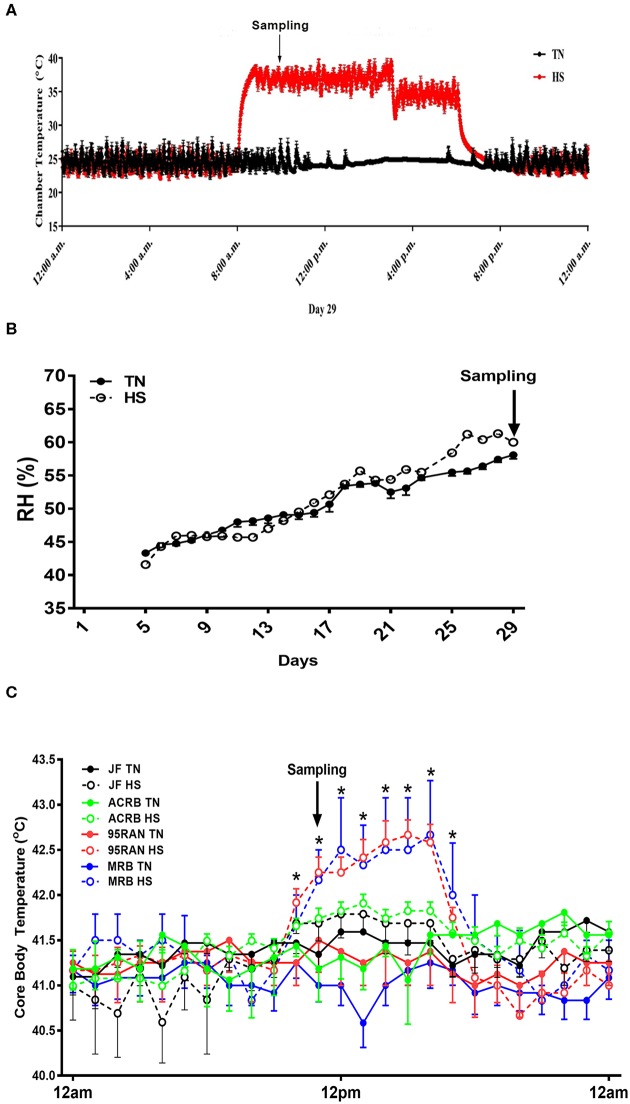
Effect of heat stress on core body temperature in modern broilers and wild JF birds. Birds were maintained in environmental chambers and exposed to two environmental (TN, 24°C vs. HS, 36°C) conditions **(A)** with an average RH of 55% **(B)**. Core body temperature was continuously monitored using thermochron temperature logger (iButton, DS1922L, Maxim, CA) **(C)**. Data are mean ± SEM (n=12). *indicates significant difference at *P* < 0.05. ACRB, Athens Canadian Random Bred; HS, heat stress; JF, jungle fowl; MRB, modern random bred; RH, relative humidity; TN, thermoneutral.

**Figure 2:**
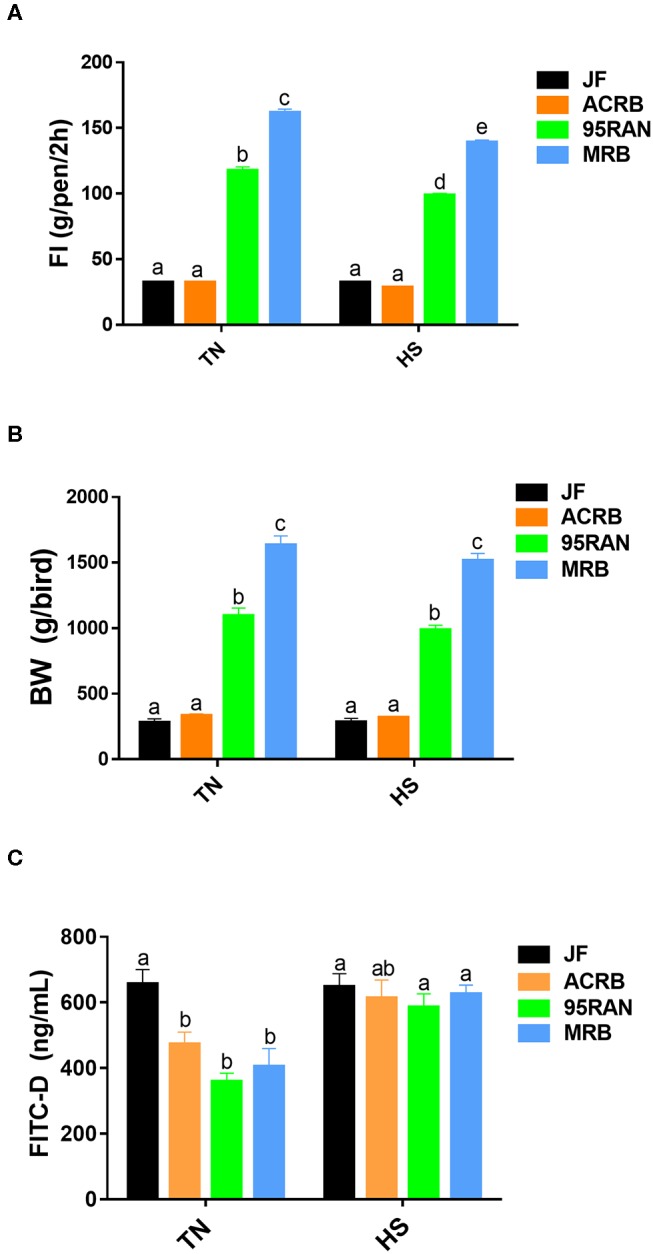
Effect of heat stress on feed intake, body weight, and serum FITC-D levels in modern broilers and wild JF birds. FI **(A)**, body weight **(B)**, and FITC-D levels **(C)** were measured. Data are presented as mean ± SEM (*n* = 75 birds/group for body weight, *n* = 3 pens/group for FI, and *n* = 6/group for FITC-D). Different letters indicate significant difference at *P* < 0.05.

As depicted in Figure 1A, environmental temperature in the experimental chambers reached 36°C within 15 min, however the RH was about 45–55% (Figure 1B). Shortly (~50 min to 1 h) after heat stress exposure, core body temperature of 95RAN and MRB chickens was significantly increased by ~0.5–1°C compared to their counterparts maintained at TN conditions (Figure 1C). The core body temperature of JF and ACRB was not affected by heat stress exposure (Figure 1C). This increase in core body temperature-induced by heat stress resulted in a significant decrease in feed intake of 95RAN and MRB, but not that of JF and ACRB (Figure 2A). Although slightly decreased, BW was not statistically affected by acute heat stress in all chicken populations (Figure 2B). Acute heat stress significantly increased serum FITC-D levels in 95RAN and MRB, but not in JF and ACRB (Figure 2C), indicating potential leaky gut syndrome.

### Effects of Heat Stress on Intestinal HSP Expression

**Figure 3:**
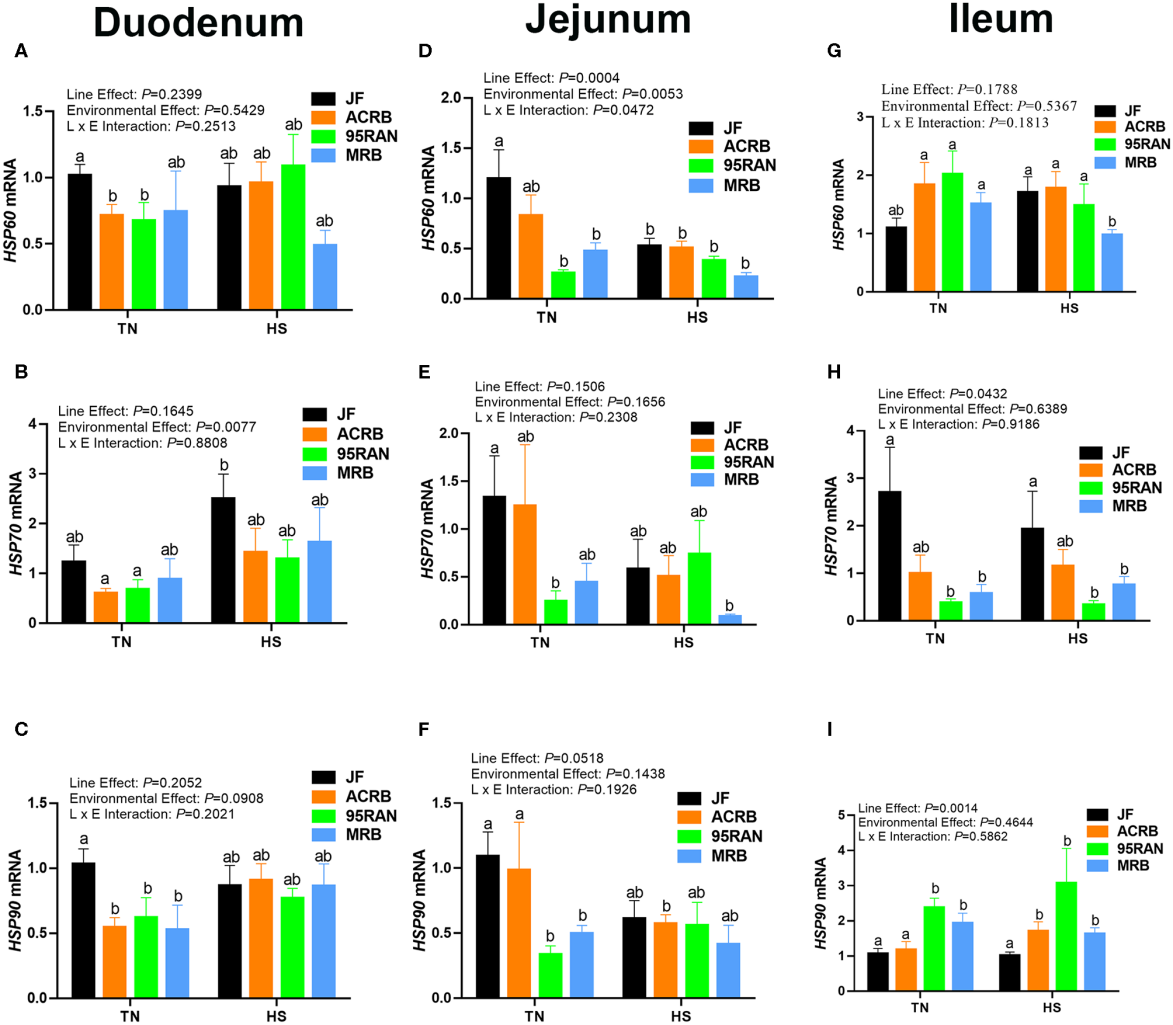
Effect of heat stress on intestinal HSP expression in modern broilers and wild JF birds. The relative expression of *HSP60, HSP70*, and *HSP90* was determined in the duodenum **(A-C)**, jejunum **(D-F)**, and ileum **(G-I)** using real-time quantitative PCR and 2^−ΔΔCt^ method (30). Data are mean ± SEM (*n* = 8/group). Different letters indicate significant difference at *P* < 0.05. ACRB, Athens Canadian Random Bred; HS, heat stress; JF, jungle fowl; MRB, modern random bred; RH, relative humidity; TN, thermoneutral.

To gain further insights, molecular analyses showed that, under TN conditions, the expression of HSP60 and HSP70 gene was significantly lower in the duodenum of ACRB and 95RAN and in the jejunum of 95RAN and MRB compared to their JF ancestor (Figures 3A,D). In the ileum, HSP70 mRNA abundance was significantly down-regulated in 95RAN and MRB however HSP60 mRNA levels did not differ between all studied populations (Figures 3G,H). The expression of HSP90 gene was significantly down-regulated in the duodenum and jejunum of all broiler populations, and it was upregulated in the ileum of 95RAN and MRB compared to the JF (Figures 3C,F,I). Acute heat stress exposure significantly down-regulated the expression of HSP60 in the jejunum of JF and in the ileum of MRB compared to their TN counterparts (Figures 3D,G). The duodenal HSP60 expression was not affected by heat stress in all bird populations (Figure 3A). Heat stress significantly up regulated the ileal HSP90 expression only in ACRB (Figure 3I), and without eliciting any changes in the expression of HSP70 and HSP90 in other intestinal segments in other populations (Figures 3B,C,E,F,H,I).

### Effects of Heat Stress on Intestinal Tight, Adherens, and Gap Junction Expression

**Figure 4:**
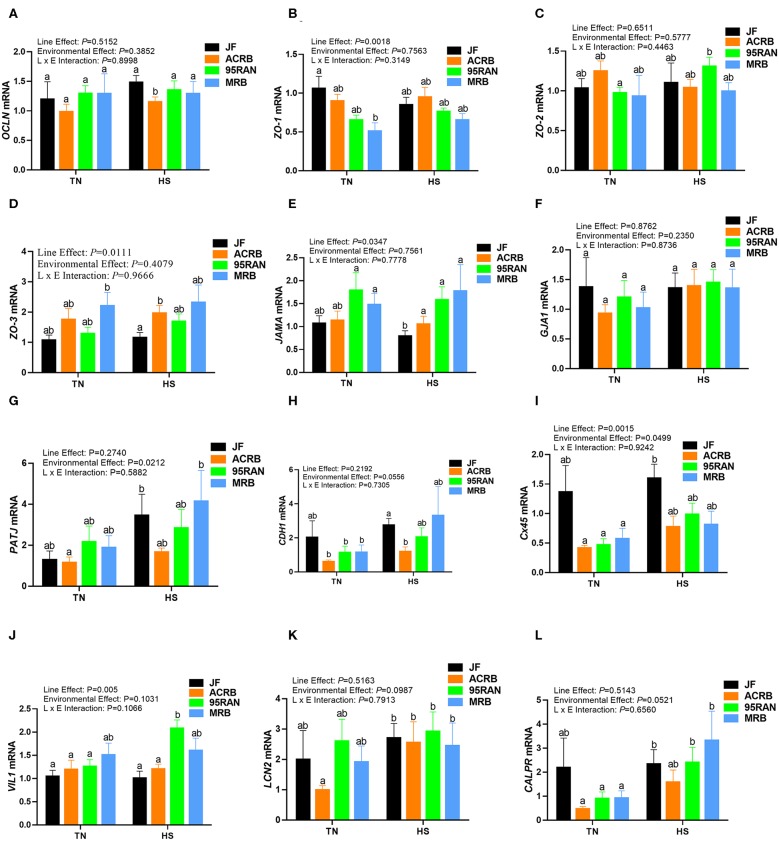
Effect of heat stress on intestinal tight junction expression in modern broilers and wild JF birds. The relative expression of *OCLN* **(A)**, *ZO-1* **(B)**, *ZO-2* **(C)**, *ZO-3* **(D)**, *JAMA* **(E)**, *GJA1* **(F)**, *PATJ* **(G)**, *CDH1* **(H)**, *Cx45* **(I)**, *VIL1* **(J)**, *LCN2* **(K)**, and *CALPR* **(L)** was determined in the duodenum using real-time quantitative PCR and 2^−ΔΔCt^ method (30). Data are mean ± SEM (*n* = 8/group). Different letters indicate significant difference at *P* < 0.05. ACRB, Athens Canadian Random Bred; CALPR, calprotectin; CDH1, cadherin 1; Cx45, connexin 45; GJA1, gap junction protein alpha 1; HS, heat stress; JAMA, junctional adhesion molecule A; JF, jungle fowl; LCN2, lipocalin 2; MRB, modern random bred; *OCLN*, occludin; PATJ, PALS1-associated tight junction protein; RH, relative humidity; TN, thermoneutral; VIL1, villin 1; ZO-1/2/3, zonula occludens.

**Figure 5:**
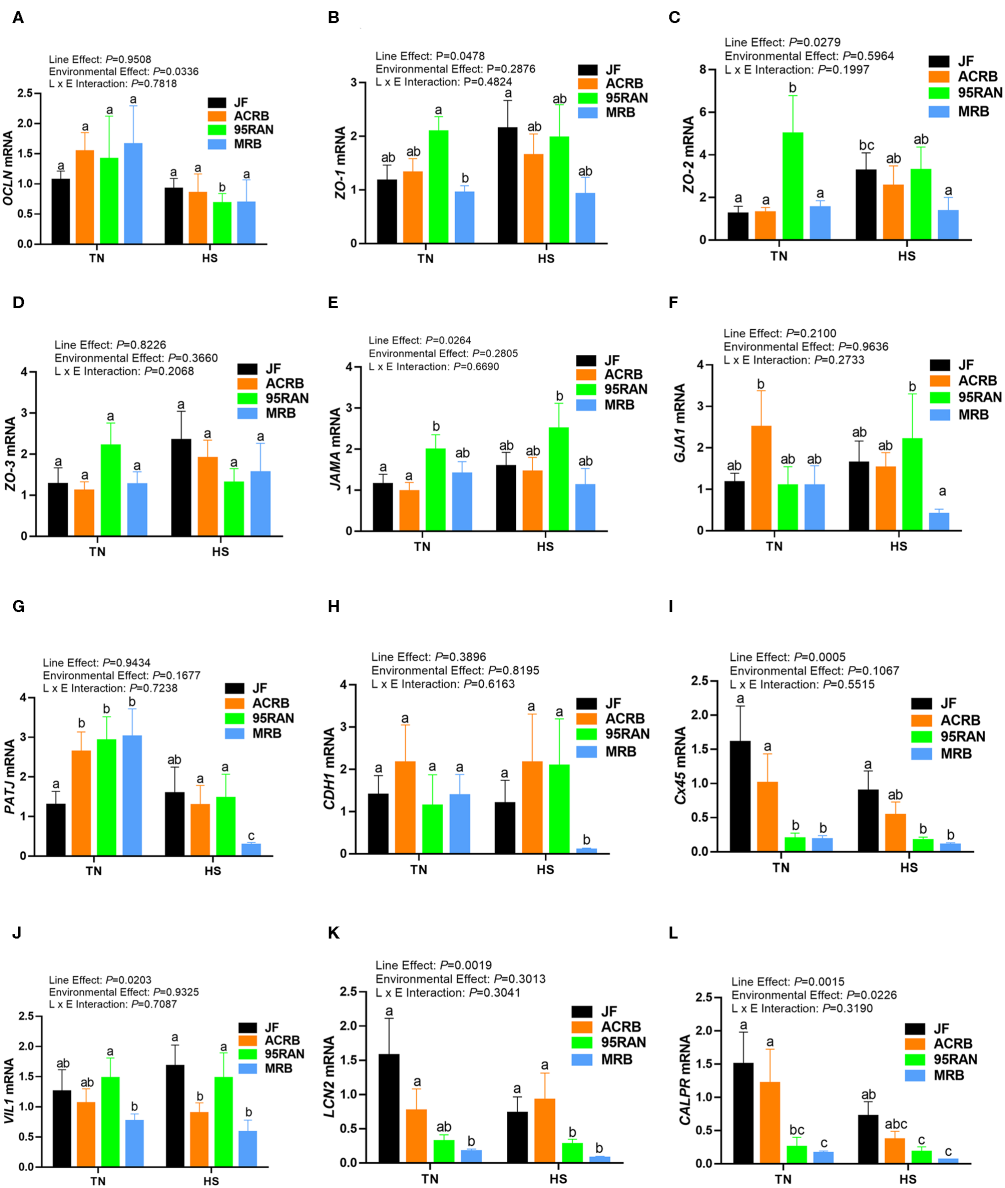
Effect of heat stress on intestinal tight junction expression in modern broilers and wild JF birds. The relative expression of *OCLN* **(A)**, *ZO-1* **(B)**, *ZO-2* **(C)**, *ZO-3* **(D)**, *JAMA* **(E)**, *GJA1* **(F)**, *PATJ* **(G)**, *CDH1* **(H)**, *Cx45* **(I)**, *VIL1* **(J)**, *LCN2* **(K)**, and *CALPR* **(L)** was determined in the jejunum using real-time quantitative PCR and 2^−ΔΔCt^ method (30). Data are mean ± SEM (*n* = 8/group). Different letters indicate significant difference at *P* < 0.05. ACRB, Athens Canadian Random Bred; CALPR, calprotectin; CDH1, cadherin 1; Cx45, connexin 45; GJA1, gap junction protein alpha 1; HS, heat stress; JAMA, junctional adhesion molecule A; JF, jungle fowl; LCN2, lipocalin 2; MRB, modern random bred; *OCLN*, occludin; PATJ, PALS1-associated tight junction protein; RH, relative humidity; TN, thermoneutral; VIL1, villin 1; ZO-1/2/3, zonula occludens.

**Figure 6:**
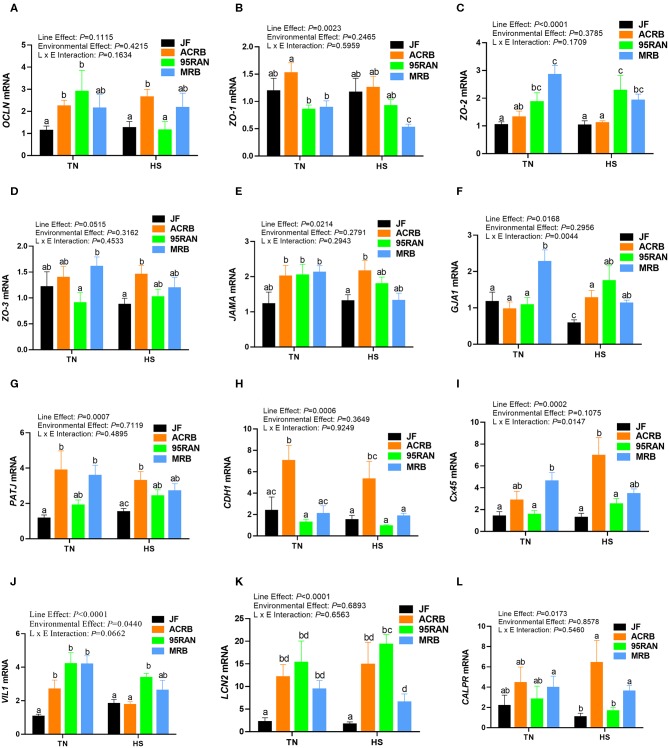
Effect of heat stress on intestinal tight junction expression in modern broilers and wild JF birds. The relative expression of *OCLN* **(A)**, *ZO-1* **(B)**, *ZO-2* **(C)**, *ZO-3* **(D)**, *JAMA* **(E)**, *GJA1* **(F)**, *PATJ* **(G)**, *CDH1* **(H)**, *Cx45* **(I)**, *VIL1* **(J)**, *LCN2* **(K)**, and *CALPR* **(L)** was determined in the ileum using real-time quantitative PCR and 2^−ΔΔCt^ method (30). Data are mean ± SEM (*n* = 8/group). Different letters indicate significant difference at *P* < 0.05. ACRB, Athens Canadian Random Bred; CALPR, calprotectin; CDH1, cadherin 1; Cx45, connexin 45; GJA1, gap junction protein alpha 1; HS, heat stress; JAMA, junctional adhesion molecule A; JF, jungle fowl; LCN2, lipocalin 2; MRB, modern random bred; *OCLN*, occludin; PATJ, PALS1-associated tight junction protein; RH, relative humidity; TN, thermoneutral; VIL1, villin 1; ZO-1/2/3, zonula occludens.

Under TN conditions, only the expression of zonula occludins 1 (*ZO-1*) was significantly lower in the duodenum of MRB compared to JF birds (Figure 4B). The duodenal expression of occludin (*OCLN*), *ZO-2, ZO-3*, junctional adhesion molecule A (*JAMA*), gap junction protein alpha 1 (*GJA-1*), PALS1-associated tight junction protein (*PATJ*), cadherin 1 (*CDH1*), connexin 45 (*Cx45*), villin 1 (*VIL1*), lipocalin 2 (*LCN2*), and calprotectin (*CALPR*) gene did not differ between all populations under TN conditions (Figures 4A–I). In the jejunum, the expression of *ZO-2* and *JAMA* was significantly up regulated in 95RAN (Figures 5C,E) and the expression of *PATJ* was up regulated in all broiler population compared to JF birds (Figure 5G). The jejunal expression of *Cx45* and *CALPR* was significantly down regulated in 95RAN and MRB, and the expression of jejunal *LCN2* was significantly decreased in MRB compared to JF population (Figures 5I,K,L). In the ileum, *VIL1* and *LCN2* expression was significantly higher in ACRB, 95RAN, and MRB compared to JF (Figures 6J,K). The ileal expression of *ZO-2, GJA1, PATJ*, and *Cx45* was significantly up regulated in MRB compared to JF birds (Figures 6C,F,G,I). Ileal *JAMA, VIL1*, and *LCN2* expression was higher (*P* < 0.05) in all broiler populations compared to JF (Figures 6E,G,J). Ileal *OCLN* expression was higher in ACRB and 95RAN (Figure 6A), and ileal *CDH1* expression was higher (*P* < 0.05) in ACRB compared to JF population (Figure 6H).

Acute heat stress exposure significantly up regulated the duodenal expression of *OCLN* and *LCN2* in ACRB, *ZO-2, VIL1*, and *CALPR* in 95 RAN, and only *CALPR* in MRB compared to their TN counterparts (Figures 4A–L). In the jejunum however, heat stress down regulated the expression of *PATJ* in ACRB, 95RAN, and MRB, and that of *CDH1* in MRB (Figures 5A–L). In the ileum, heat stress significantly down regulated the expression of *OCLN* in 95 RAN, *ZO-1* in MRB, *GJA* in JF, and *VIL1* in ACRB compared to their TN counterparts (Figures 6A–L).

## Discussion

The development of genetically superior broilers capable of higher productivity and higher economic efficiency in a short period of time has transformed poultry from rural farming to full-sophisticated industry within ~70 years. In the present study, the 4- to 6-fold increase in body weight of 29 d-old broilers from the 1990's (95RAN) and 2015 (MRB) compared to the broilers from 1950's (ACRB) and the ancestral wild-type JF illustrated the above mentioned progresses. These genetic lines represent extremely valuable research resources for intraspecific comparative studies.

Associated with these astounding genetic progresses, there was a number of unattended undesirable changes including high sensitivity to high environmental temperature (33). Large, abrupt, and widespread extreme heat waves have occurred repeatedly in the past (7), and predicted to increase for the next century (8). In modern broiler chickens, which supports the livelihoods and food security of billions of people (5), the strong negative effect of heat stress on growth, feed efficiency, meat yield and mortality is well documented (10–18), and such effects will take a heavy toll during the next decades as the distribution of heat anomalies continues to increase (9). Aforesaid effects will inflict heavy economic losses to the poultry industry (34). Depressed feed intake observed here and in many previous studies (13, 33, 35) is very likely the starting point and the most prominent effect of heat stress. Interestingly, in our experimental conditions, feed intake was reduced only in 95RAN and MRB, but not in ACRB and JF populations indicating that modern broilers are more senstitive to heat stress than their counterparts of the 1950's (ACRB) and the ancestral wild JF. This could be associated with higher metabolic rate and heat production in modern broilers (36), which was evidenced here by a significant increase in core body temperature of 95RAN and MRB but not that of ACRB and JF. In fact, in order to reduce diet-induced thermogenesis and body heat production, broilers reduced their feed intake and divert blood to areas near the skin to dissipate the excess of heat during high environmental temperature. The reduced energy intake combined with reduced blood and oxygen supplies can result in multiple organ damage, and in extreme cases can lead to death.

Gastrointestinal tract, which provides the biological environment for nutrient digestion and absorption as well as a protection against pathogens and toxins, has been recently shown to be one target for heat stress damage (37, 38). Marchini and co-workers have shown that heat stress altered broiler intestinal morphometry (21). Quinteiro-Filho et al. reported that heat stress induced intestinal injury and altered macrophage activity in broiler chickens (39). In a recent study, Dridi's group has shown that heat stress increase FITC-D levels in chicken serum indicating an increased intestinal permeability and leacky gut syndrome (22). In support of these data, circulating FITC-D levels were significantly increased in heat-stressed 95RAN and MRB populations but not in ACRB and JF compared to their TN counterparts. Intriguingly, FITC-D levels were higher in JF compared to other populations under TN conditions. The mechanisms behind this enigma are not known at this time and merit further in depth investigations.

The intestinal epithelial barrier is a one-cell-thick internal lining of the gut that contains different types of epithelial cells. A pivotal function of these cells is the maintenance of barrier integrity which allows selective permeability of essential ions, nutrients, and water but restricts the entry of bacterial toxins and pathogens (40). This physical barrier is facilitated by tight connections between each cell. A number of tight junction protein such as claudin, occludin, and ZO seal the paracellular pathway and conduct gate and fence functions. Here, the expression of tight junction, gap junction, and adherens junction genes is environment-, genotype-, and intestinal segment-dependent. For instance, heat stress exposure up regulated the duodenal expression of *OCLN* and *LCN2* in ACRB, *ZO-2, VIL1*, and *CALPR* in 95 RAN, and *CALPR* in MRB. In the jejunum however, heat stress down regulated the expression of *PATJ* in ACRB, 95RAN, and MRB, and that of *CDH1* in MRB. In the ileum, heat stress down regulated the expression of *OCLN* in 95 RAN, *ZO-1* in MRB, *GJA* in JF, and *VIL1* in ACRB. Although the upstream mediators and the mechanisms behind these differential regulations are not known at this time, it is possible that the induction of *OCLN* and *LCN2* expression in the ACRB duodenum play a protective response against heat stress. In support of this speculation, previous studies have shown that thermal stress induce *OCLN* and *LCN2* expression (41, 42) and that overexpression of *OCLN* enhanced tight junction barrier function in MDCK cells (43). Conversely, *OCLN* knock down led to epithelial cell alteration and an increased intestinal permeability to selected paracellular markers (44). Similarly, Li and Mrsny (45) demonstrated that oncogenic Raf-1 disrupted epithelial tight junctions via *OCLN* downregulation. Roudkenar and co-workers have shown that ectopic expression of *LCN2* protected CHO and HEK293 cells from heat stress (42). The high expression of *CALPR* in the MRB duodenum, on the other hand, suggested an inflammation state (46) and an increased intestinal permeability induced by heat stress which is supported in our experimental conditions by higher serum concentrations of FITC-D. Furthermore, the down regulation of jejunal *CDH1* and ileal *ZO-1* expression continue to support that heat stress induce intestinal epithelial barrier dysfunction in MRB birds (47–50).

As heat shock response is a major cellular stress pathway that is activated in response to heat stress,we sought to determine next the expression of heat shock proteins. As for the tight junction proteins, the expression of HSPs is environment-, genotype-, and intestinal segment-dependent. Although and unexpectedly, heat stress did not elicit any changes except a down regulation of HSP60 mRNA in MRB ileum and HSP90 mRNA in ACRB jejunum, the expression of HSPs differ between genotypes under TN conditions. In fact, the expression of jejunal HSP60/70/90 and ileal HSP70 was lower, but the expression of ileal HSP90 was higher in modern (95RAN and MRB) compared to JF under TN conditions. The absence of HSP mRNA induction by heat stress might be explained by quick mRNA degradation, turnover, or half lives and short intrinsic stability and decay (51, 52). Further studies measuring protein levels of both HSPs and tight junction proteins are warranted to better delineate the mechanisms emplyed by heat stress to alter intestinal barrier integrity.

In summary, this is the first report, to our knowledge, showing the effect of heat stress on gut integrity in three broiler populations characterized by different growth (slow, moderate, and rapid) rates and in their ancestor wild JF birds. The study identified several potential key molecular signatures that need further in depth investigation to better understand heat stress response in broiler chickens.

## Data Availability Statement

All datasets generated for this study are included in the article/supplementary material.

## Ethics Statement

The animal study was reviewed and approved by the University of Arkansas Animal Care and Use Committee under protocols 18083 and 16084.

## Author Contributions

SD conceived and designed the study and wrote the paper with a critical review by all authors. TT, EG, SO, JH, NA, and SD conducted the experiments. TT analyzed the data.

## Conflict of Interest

The authors declare that the research was conducted in the absence of any commercial or financial relationships that could be construed as a potential conflict of interest.
